# Molecular Defects in Cardiac Myofilament Ca^2+^-Regulation Due to Cardiomyopathy-Linked Mutations Can Be Reversed by Small Molecules Binding to Troponin

**DOI:** 10.3389/fphys.2018.00243

**Published:** 2018-03-27

**Authors:** Alice Sheehan, Andrew E. Messer, Maria Papadaki, Afnan Chaudhry, Vladimír Kren, David Biedermann, Brian Blagg, Anuj Khandelwal, Steven B. Marston

**Affiliations:** ^1^NHLI, Imperial College London, London, United Kingdom; ^2^Laboratory of Biotransformation, Institute of Microbiology of the Czech Academy of Sciences, Prague, Czechia; ^3^Department of Medicinal Chemistry, The University of Kansas, Lawrence, KS, United States

**Keywords:** cardiomyopathy, sarcomeric protein mutations, troponin I phosphorylation, PKA, Ca^2+^ regulation, small molecule pharmacology, EGCG, silybin

## Abstract

The inherited cardiomyopathies, hypertrophic cardiomyopathy (HCM) and dilated cardiomyopathy (DCM) are relatively common, potentially life-threatening and currently untreatable. Mutations are often in the contractile proteins of cardiac muscle and cause abnormal Ca^2+^ regulation *via* troponin. HCM is usually linked to higher myofilament Ca^2+^-sensitivity whilst in both HCM and DCM mutant tissue there is often an uncoupling of the relationship between troponin I (TnI) phosphorylation by PKA and modulation of myofilament Ca^2+^-sensitivity, essential for normal responses to adrenaline. The adrenergic response is blunted, and this may predispose the heart to failure under stress. At present there are no compounds or interventions that can prevent or treat sarcomere cardiomyopathies. There is a need for novel therapies that act at a more fundamental level to affect the disease process. We demonstrated that epigallocatechin-3 gallate (EGCG) was found to be capable of restoring the coupled relationship between Ca^2+^-sensitivity and TnI phosphorylation in mutant thin filaments to normal *in vitro*, independent of the mutation (15 mutations tested). We have labeled this property “re-coupling.” The action of EGCG *in vitro* to reverse the abnormality caused by myopathic mutations would appear to be an ideal pharmaceutical profile for treatment of inherited HCM and DCM but EGCG is known to be promiscuous *in vivo* and is thus unsuitable as a therapeutic drug. We therefore investigated whether other structurally related compounds can re-couple myofilaments without these off-target effects. We used the quantitative *in vitro* motility assay to screen 40 compounds, related to C-terminal Hsp90 inhibitors, and found 23 that can re-couple mutant myofilaments. There is no correlation between re-couplers and Hsp90 inhibitors. The Ca^2+^-sensitivity shift due to TnI phosphorylation was restored to 2.2 ± 0.01-fold (*n* = 19) compared to 2.0 ± 0.24-fold (*n* = 7) in wild-type thin filaments. Many of these compounds were either pure re-couplers or pure desensitizers, indicating these properties are independent; moreover, re-coupling ability could be lost with small changes of compound structure, indicating the possibility of specificity. Small molecules that can re-couple may have therapeutic potential.

**HIGHLIGHTS**

- Inherited cardiomyopathies are common diseases that are currently untreatable at a fundamental level and therefore finding a small molecule treatment is highly desirable.
- We have identified a molecular level dysfunction common to nearly all mutations: uncoupling of the relationship between troponin I phosphorylation and modulation of myofilament Ca^2+^-sensitivity, essential for normal responses to adrenaline.
- We have identified a new class of drugs that are capable of both reducing Ca^2+^-sensitivity and/or recouping the relationship between troponin I phosphorylation and Ca^2+^-sensitivity.
- The re-coupling phenomenon can be explained on the basis of a single mechanism that is testable.
- Measurements with a wide range of small molecules of varying structures can indicate the critical molecular features required for recoupling and allows the prediction of other potential re-couplers.

## Introduction

The contractile apparatus of cardiac muscle is controlled by the sarcoplasmic Ca^2+^-level acting upon the Ca^2+^ sensor, troponin. Troponin, together with tropomyosin and actin constitutes the thin filaments of muscle. In unstimulated muscle contraction is inhibited by the C-terminus of the troponin I subunit binding to actin-tropomyosin and blocking the interaction with myosin crossbridges. Upon stimulation intracellular Ca^2+^ rises and Ca^2+^ binds to the troponin C subunit (TnC), the C-terminus of TnI is released from its site on actin and crossbridge cycling can proceed (Gordon et al., 2000; MacLeod et al., 2010). Thus changes in Ca^2+^ level switch contraction on and off. However contractility in heart muscle is additionally modulated through the β-receptors. Activation of β1- receptors by adrenaline initiates formation of cAMP and activation of Protein Kinase A (PKA) leading to increased rate and magnitude of contraction. Lusitropy, an increased rate of relaxation allowing for shorter twitch duration, is a key component of adrenergic activation and involves TnI. Phosphorylation of ser22 and 23 of TnI by PKA leads to a 2-fold decrease in Ca^2+^-sensitivity and a corresponding increase in the rate of Ca^2+^ release from TnC and is essential for the lusitropic response (Robertson et al., 1982; Kentish et al., 2001; Layland et al., 2005).

Whilst the structural mechanism of the troponin Ca^2+^ switch is well-documented (Takeda et al., 2003), the structural basis of lusitropy is poorly understood since both the phosphorylated N-terminal peptide and the regulatory “switch peptide” of TnI are intrinsically disordered, however recent studies using molecular dynamics simulations have begun to explain this phenomenon (Papadaki and Marston, 2016). The study of this phosphorylation dependent regulation has been recently stimulated by the discovery that a primary effect of many mutations in thin filament proteins associated with cardiomyopathy is the uncoupling of this relationship (Bayliss et al., 2012; Memo et al., 2013; Messer and Marston, 2014).

Dilated cardiomyopathy (DCM) and hypertrophic cardiomyopathy (HCM) are often due to single mutations in a protein of the sarcomere and have been extensively studied *in vitro*. It is estimated that 1/250 of the population show symptoms of DCM, and that 40% of cases are inherited (Hershberger et al., 2013). Truncating mutations in the titin (TTN) gene are the most common but a substantial fraction (12%) of mutations are in the proteins of the thin filament. HCM is estimated to be present in 1/500 of the population; it is overwhelmingly an inherited disease and the majority of known disease causing mutations are in the contractile proteins of the heart muscle sarcomere (Walsh et al., 2017). It is a leading cause of sudden cardiac death in young adults, particularly athletes and if not controlled can lead to heart failure (Lopes and Elliott, 2014).

In HCM, mutations generally cause an increase of about 1.5- to 2-fold in myofilament Ca^2+^-sensitivity that is considered to be sufficient to induce the symptoms of HCM. The effect of DCM mutations in contractile proteins on Ca^2+^-sensitivity is variable with a trend toward deceased Ca^2+^-sensitivity (Marston, 2016). Importantly for this study it has been shown that HCM and DCM-related mutations in contractile proteins usually abolish the coupled relationship between Ca^2+^-sensitivity and TnI phosphorylation by PKA: we have called this phenomenon “uncoupling” (Memo et al., 2013; Messer and Marston, 2014; Messer et al., 2016). A blunting of the response to β-adrenergic activation is commonly observed in animal models with HCM or DCM mutations and it has been demonstrated in a DCM mouse model that this blunting is sufficient to induce heart failure under chronic stress (Messer and Marston, 2014; Wilkinson et al., 2015).

At present there are no compounds or interventions that can prevent or treat sarcomeric cardiomyopathies and current treatments available are directed at alleviating the symptoms. There is a need for novel therapies that act at a more fundamental level to affect the disease process (Tardiff et al., 2015). In our studies of the uncoupling effects of mutations we demonstrated that epigallocatechin-3 gallate (EGCG), originally studied as a Ca^2+^ desensitizer (Tadano et al., 2010; Friedrich et al., 2016), was also found to be capable of restoring the coupled relationship between Ca^2+^-sensitivity and TnI phosphorylation in mutant thin filaments to normal *in vitro*, a property we refer to as “re-coupling” (Papadaki et al., 2015; Messer et al., 2016, 2017). The action of EGCG *in vitro* to reverse the abnormality caused by myopathic mutations would appear to be an ideal pharmaceutical profile for treatment of inherited HCM and DCM. However, EGCG is known to have pleiotropic pharmaceutical properties in intact tissue, including inotropic activity, making it an unsuitable therapeutic drug (Singh et al., 2011; Feng et al., 2012; Baell and Walters, 2014; Ingólfsson et al., 2014). Moreover, this compound is not easily available in its pure state and it is not sufficiently stable under oxidative and hydrolytic conditions. Therefore, it is important to investigate whether other structurally related compounds can also re-couple myofilaments without these off-target effects.

EGCG and its analogs have been widely studied as Hsp90 inhibitors, so we began our investigations using analogs of EGCG and of Silybin, a natural product with structural similarity, originally studied as Hsp90 inhibitors (Hao et al., 2010; Zhao et al., 2011; Khandelwal et al., 2013). We used the quantitative *in vitro* motility assay to screen 40 compounds and found 23 that can re-couple mutant myofilaments. Many of these compounds were either pure re-couplers or pure desensitizers, unlike EGCG that has both properties. Moreover, re-coupling ability could be lost with minor modifications in the compound structure, indicating the possibility of specificity. Consideration of the molecular structures of re-coupling molecules (structure-activity relationships, SAR), compared with similar inactive molecules can provide considerable insight into the mechanism of re-coupling and may lead to the discovery of more potent re-coupling (lead) compounds with therapeutic potential.

## Methods

### Sources of contractile proteins

Troponin was isolated from donor heart tissue, supplied by Sydney Tissue Bank, University of Sydney, Australia. St Vincent's Hospital Sydney and Brompton, Harefield and NHLI Research Ethics Committees provided ethical approval for the collection of and experimentation with tissue samples (Lal et al., 2015). Donors had no previous history of heart disease and unremarkable ECG.

An anti-cardiac TnI monoclonal antibody affinity column was used to isolate troponin from 100 mg of donor heart tissue as previously described (Messer et al., 2007). This troponin has an intrinsically high level of TnI phosphorylation (1.6–2.2 mol P_i_/mol). To reduce the phosphorylation level (<0.3 mol P_i_/mol) troponin was treated with shrimp alkaline phosphatase (Memo et al., 2013). The level of TnI phosphorylation was measured by phosphate affinity SDS–PAGE as previously described (Messer et al., 2009).

Recombinant human sequence tropomyosin was a gift from Kristen Nowak and Elyshia MacNamara, University, Western Australia. Wild-type α-tropomyosin (Tpm1.1) and mutant E180G tropomyosin were expressed in a baculovirus/Sf9 system (Akkari et al., 2002; Marston et al., 2013). Skeletal muscle F-actin and myosin were isolated from rabbit fast-twitch skeletal muscle as previously described (Messer et al., 2007). TnC G159D mutant troponin was extracted from a mutant human heart sample (Dyer et al., 2009), E99K mutant cardiac actin was extracted from the E99K transgenic mouse heart (Song et al., 2013).

### EGCG- and silybin-related compounds

The analogs of EGCG labeled **1**–**28** were prepared as described by Khandelwal and Blagg (Khandelwal et al., 2013). Table 1 identifies the compounds in that paper and the molecular structures are shown in Figure S1. Silybin diastereomereomers and silmarin-derived pure flavonolignanswere isolated as described by Kren et al. (Gažák et al., 2011; Krenek et al., 2014; Novotná et al., 2014) (for structures see Figure S1). All other compounds were purchased from Sigma-Aldrich. Dose was 100 μM from a 10 mM stock in DMSO unless otherwise stated.

**Table 1:** Ability of small molecules to re-couple troponin in thin myofilaments measured by *in vitro* motility assay.

**Drug**	**Coupling Difference (Initial Screen E180G)**	**E180G EC_50_ % Motility Difference, μM**	**Other mutations**	**EC_50_ Hsp90 inhibition assay**
EGCG	23.0	59 μM	see Figure 1	
Silybin(racemic mixture)	12.0	47 μM	E54K, E99K	
1	1.0			
2 (23h)	27.0	30 μM		22 μM
3 (27a)	8.0			14 μM
4 (23p)	27.0	34 μM		>100 μM
5	2.0			
6	1.0			
7 (27c)	−11.0	67 μM	E54K, E99K	32 μM
8 (27p)	No Effect			38 μM
9 (23q)	20.0	35 μM		>100 μM
10	No Effect			
11 (10c)	7.0		E54K	>100 μM
12 (23f)	−1.0	No Effect		63 μM
13	No Effect			
14	17.0	36 μM		
15 (10j)	13.0			19 μM
16 (27h)	6.0	31 μM		8 μM
17 (23b)	3.5			58 μM
19 (27g)	24.0			21 μM
20	2.0			
21 (11d)	25.0			14 μM
22	7.0			
23 (23a)	9.0			98 μM
24	8.0	24.5 μM		
26 (7a)	26.0		E54K	
27 (31d)	4.0			55 μM
28 (10c)	4.0			>100 μM
Epicatechin (EC)	69			
Epigallocatechin (EGC)	62			
Gallocatechin (GC)	68			
Epicatechin Gallate (ECG)	8			
Silybin A	5		G159D	
Silybin B	55	39 μM	G159D	
Dehydrosilybin A	3		G159D	
Dehydrosilybin B	62	47 μM	G159D	
Isosilybin	4			
Silychristin	35			
Silydianin	20			
Taxifolin	60			
Quercetin	51	8 μM		
Resveratrol	63			
Novobiocin	46			
Pterostilbene	50			
Sissotrin	30			
W-7	50			
Omecamtiv Mecarbil	0			

*Test system as shown in Figure 2. Compounds **1–28** were synthesized by Blagg and Khandelwal; their corresponding identification numbers in the original paper are given in brackets and the structures are shown in the supplement (Khandelwal et al., 2013). Difference between motility (%) in phosphorylated and unphosphorylated forms of thin filaments (and EC_50_, when measured) with TPM1 E180G HCM mutation are shown. Other mutations also used in this test are E54K, TPM1 E54K DCM mutation; E99K, ACTC E99K HCM mutation and G159D, TNNC1 G159D DCM mutation. Compounds highlighted in green are effective re-couplers. Compounds highlighted in red indicate reverse re-coupling where Ca^2+^-sensitivity of the phosphorylated Tn I is greater than that of the dephosphorylated Tn I. In orange is the reverse re-coupler #7. For comparison the last column shows the effectiveness of these compounds in an assay for Hsp90 inhibition; effective Hsp90 inhibitors are highlighted in green and EC_50_ is given when measured (Khandelwal et al., 2013)*.

### Quantitative *in vitro* motility assay (IVMA)

Thin filaments were reconstituted with 10 nM rabbit skeletal or mouse cardiac muscle α-actin (labeled with TRITC phalloidin), tropomyosin (40–60 nM), and troponin (60 nM) to study Ca^2+^-regulation of filament motility by the quantitative *in vitro* motility assay (IVMA) (Fraser and Marston, 1995; Messer et al., 2007). Thin filament movement over a bed of immobilized rabbit fast skeletal muscle heavy meromyosin (100 μg/ml) was compared in paired motility cells, in which troponin varied by a single factor (mutation, phosphorylation state, or treatment with drug). The temperature was set to 29°C. Filament movement was recorded and analyzed as previously described (Marston et al., 1996), yielding two parameters, the fraction of filaments moving and the speed of moving filaments. Only the fraction of filaments motile is used in this study.

The fraction motile was measured over a range of Ca^2+^ concentrations to generate Ca^2+^-activation curves. The data were fitted to the 4-variable Hill equation to yield a value for EC_50_ and n_H_. Most assays of the effect of compounds are single measurements. When appropriate EC_50_ values from replicate experiments were analyzed by paired *t*-test since the distribution of EC_50_ has been shown to be normal. Error bars and n values are given in tables and figures.

For drug screening, motility was measured with paired phosphorylated and unphosphorylated tropomyosin E180G mutant-containing thin filaments at a fixed 0.037 μM Ca^2+^. Most assays were single measurements.

## Results

### EGCG can re-couple every uncoupling mutation tested

**Figure 1:**
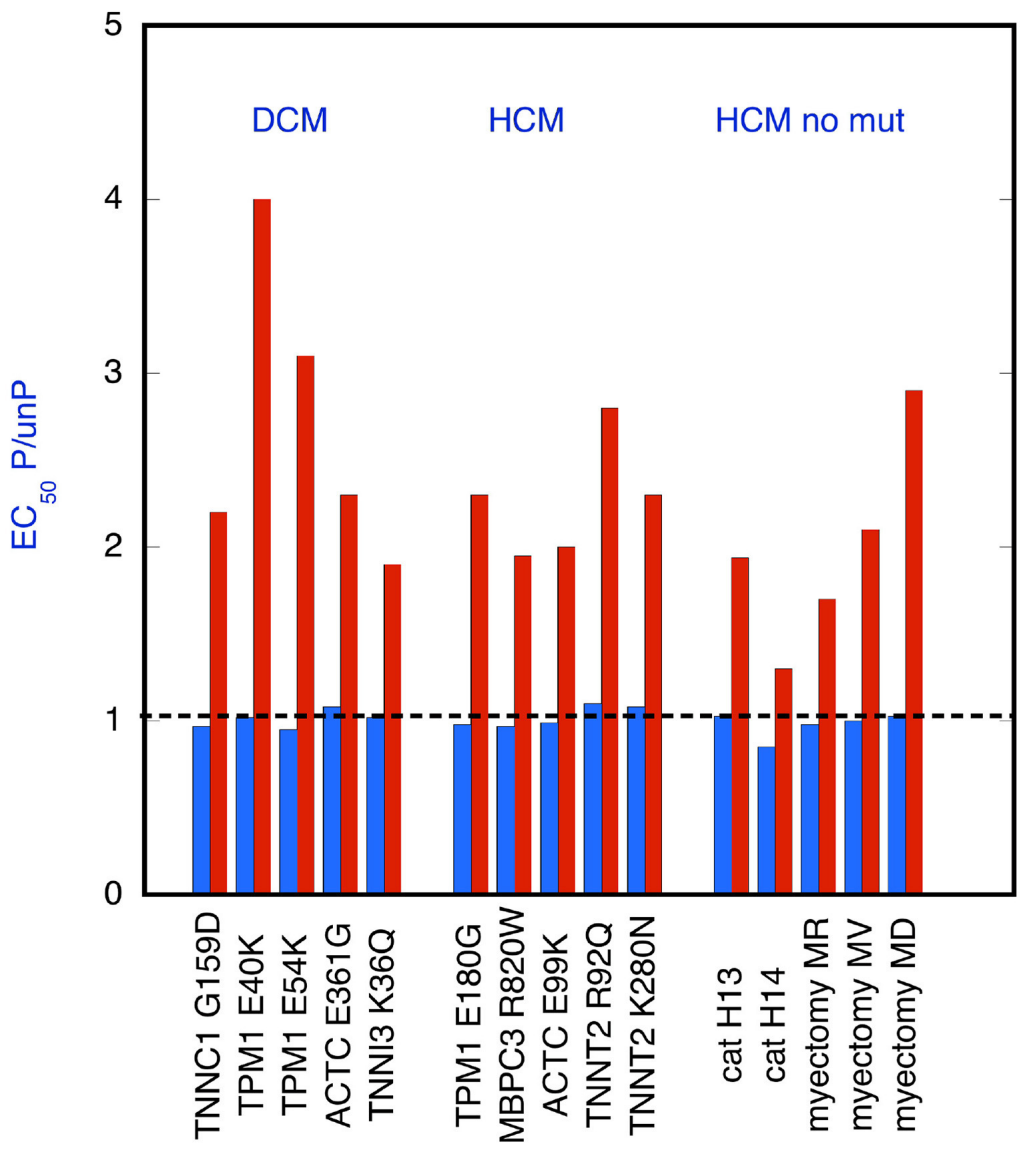
EGCG recoupling of troponin. Ratio of EC_50_ for phosphorylated and unphosphorylated thin filaments is plotted for thin filaments that are uncoupled. EC_50_ was determined from Ca^2+^-concentration plots; see Figures 2A, 4A,C,D for examples. Raw EC_50_ data is given in Table S1. EC_50_ was measured by *in vitro* motility assay in the presence (red bars) and absence (blue bars) of 100 μM EGCG. Mutant troponin or tropomyosin was from tissue (mouse, cat or human) with mutations in TnC (TNNC1), TnI (TNNI3), TnT (TNNT2), Actin (ACTC), tropomyosin (TPM1), myosin binding protein C (MYBPC3), and from cat heart with HCM and Human heart material from myectomy operations to relieve HCM; in the latter two cases samples were genotyped but no causative mutation was found. EGCG restored coupling to all the uncoupled systems. Includes data from (Papadaki et al., 2015; Messer et al., 2016, 2017).

We measured the Ca^2+^ activation of contractility using an *in vitro* motility assay that measures the movement of reconstituted thin filaments over a bed of immobilized myosin heads. A series of measurements, some of which have been previously published (Papadaki et al., 2015; Messer et al., 2016, 2017), are summarized in Figure 1. These determinations of EC_50_ show that in every case where a mutation causes uncoupling of Ca^2+^-sensitivity from TnI phosphorylation, EGCG can restore the coupling (re-couple). Thus, the effect of EGCG is independent of the mutation that induced uncoupling; in addition, both in man and cat, HCM cases were found where no mutation in sarcomeric genes was detected yet the thin filaments were uncoupled and they could also be re-coupled by EGCG (Bayliss et al., 2012; Messer et al., 2016).

### Rapid screening for re-coupling functionality

**Figure 2:**
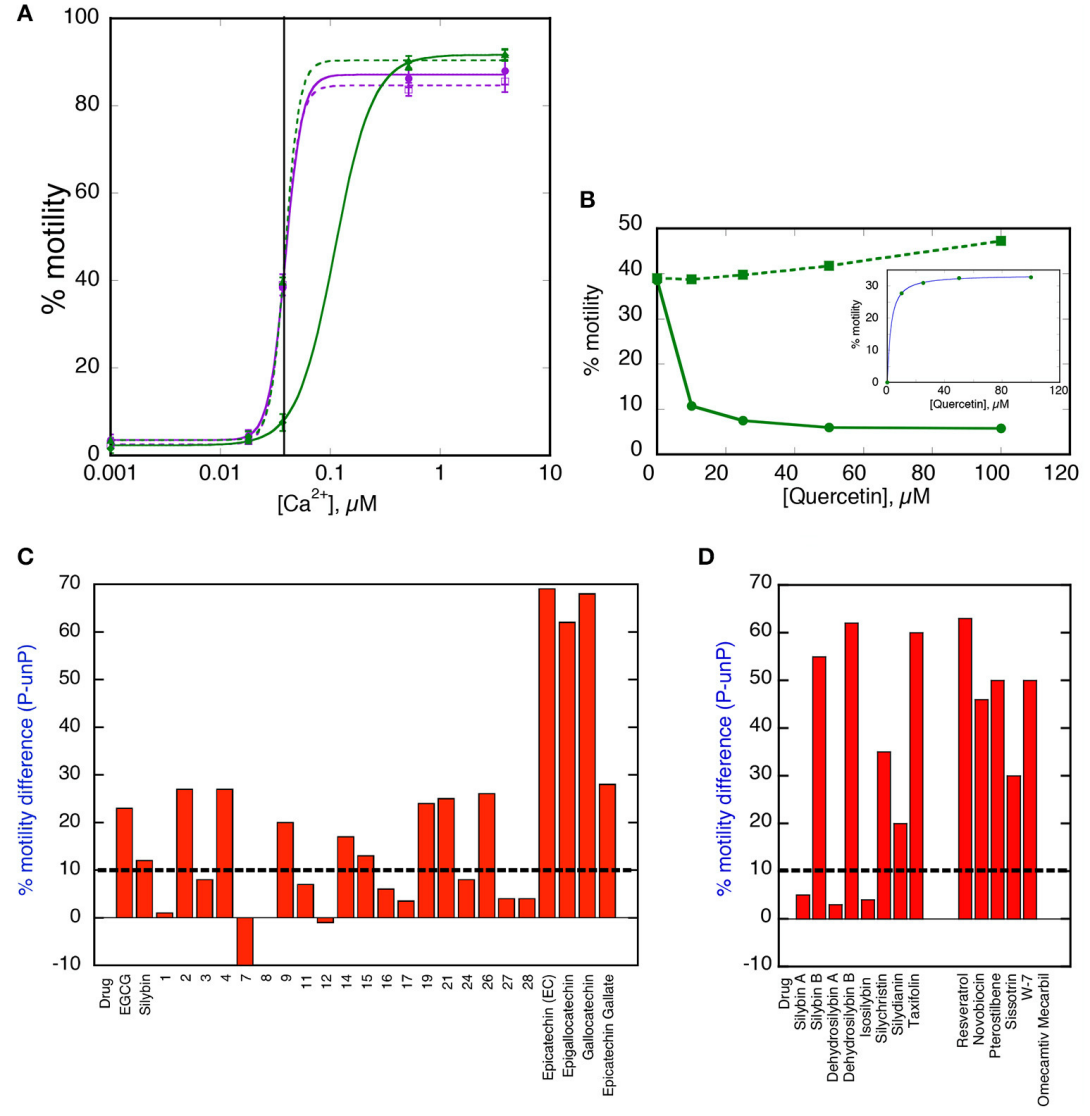
Single [Ca^2+^] screen for re-coupling compounds. **(A)** Ca^2+^-activation curves for phosphorylated and unphosphorylated thin filaments containing E180G tropomyosin in the presence and absence of 25 μM Quercetin, a typical pure recoupler. Solid lines, phosphorylated state; dotted lines, unphosphorylated state. Purple lines and points, E180G thin filaments; green lines and points, E180G thin filaments + Quercetin **(B)** Dose -response curve for quercetin recoupling activity measured at a constant concentration of 0.037 μM Ca^2+^. INSET, kinetic analysis of dose response curve; difference between phosphorylated and unphosphorylated states is plotted against quercetin concentration. The Hill equation is fitted to the data, EC_50_ = 8 μM. **(C,D)** The effect of 100 μM drug treatment on the difference between % of motility of phosphorylated and unphosphorylated tropomyosin E180G mutant-containing thin filaments at 0.037 μM Ca^2+^. **(C)** EGCG-related molecules (for structures 1–28 see Figure S1 and Table 1). **(D)** Silybin, related compounds and other HsP90 inhibitors and Ca^2+^ desensitizers. The threshold for positive re-coupling was set at a 10% difference (dotted line).

For the rapid screening of compounds, we devised a single Ca^2+^ concentration assay. This is illustrated in Figures 2A,B for quercetin. The test system contains skeletal muscle actin, human sequence recombinant tropomyosin 1.1 containing the E180G HCM-associated mutation and human cardiac troponin from donor hearts in the phosphorylated and unphosphorylated form. At 0.037 μM Ca^2+^ the filaments are about 40% motile irrespective of phosphorylation level whilst when quercetin is added the phosphorylated Ca^2+^ activity curve shifts to the right and % motility at 0.037 μM Ca^2+^ drops, whilst the unphosphorylated level is unchanged. We have set a difference between phosphorylated and unphosphorylated >10% as indicating re-coupling activity. This assay method can also be used to construct a dose-response curve as shown. Table 1 and Figures 2C,D show the results from screening 40 compounds in this assay. We investigated compounds in 4 categories.

1. Variants of EGCG lacking the pyrogallol ring (Khandelwal et al., 2013).
2. Variants of EGCG lacking the galloyl moiety (Tadano et al., 2010).
3. Silybin, its derivatives and stereoisomers, since this has also been proposed as an Hsp90 C-terminal inhibitor (Gažák et al., 2011; Zhao et al., 2011).
4. Unrelated Hsp90 inhibitors and Ca^2+^-desensitizers (Hall et al., 2014).

For structures see Figure S1.

Re-couplers were clearly distinguishable from inactive compounds: 23/40 compounds were classed as re-couplers. A series of dose-response curves (Figure S2) indicate the magnitude of recoupling is approximately the same for all compounds measured together and the EC_50_ for EGCG and Silybin derivatives are similar, being in the range 30–60 μM (see Table 1). It should be noted that Ca^2+^-sensitisers and the myosin activator Omecamtiv Mercarbil do not re-couple (Figure 2).

**Figure 3:**
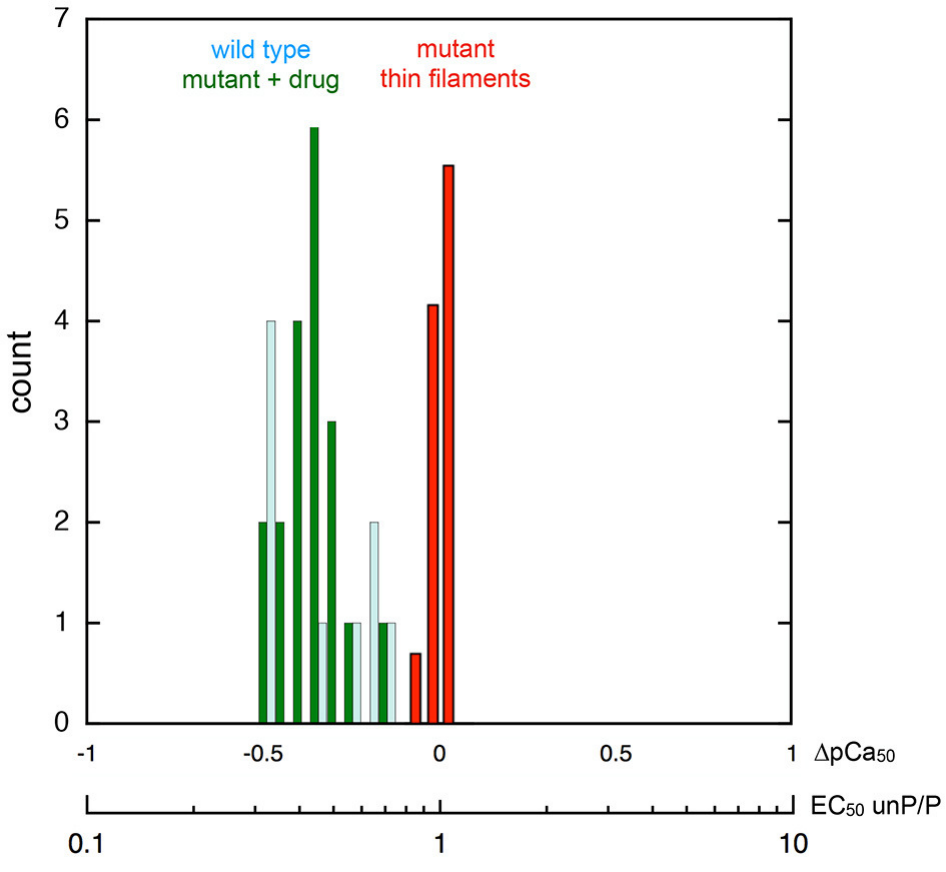
Phosphorylation-dependent change in thin filament Ca^2+^-sensitivity. Frequency histogram showing the distribution of the ratio of Ca^2+^-sensitivity in the phosphorylated and unphosphorylated state. The X-axes shows the ratio of EC_50_ unphosphorylated/phosphorylated and the corresponding shift in pCa_50_. Collected data from different mutations (Figure 1) and different recoupling compounds (Figure 2) is plotted. Red bars show distribution of shift in Ca^2+^ sensitivity on phosphorylation for mutant thin filament samples (*n* = 22 measurements by *in vitro* motility assay) showing no difference in Ca^2+^ sensitivity (uncoupling). Dark green bars show distribution of Ca^2+^ sensitivity shift from the same samples following the addition of small molecule recouplers. For comparison, the Ca^2+^-sensitivity shift on phosphorylation of native thin filaments and skinned muscle using a range of measurement systems is plotted (pale blue bars; *n* = 9, data from Marston, 2016, Table 5).

Interestingly, one compound, #**7**, had a reverse effect—the phosphorylated troponin was more Ca^2+^-sensitive than the unphosphorylated troponin condition. The standard assay used E180G mutant tropomyosin as the uncoupling mutation but, as with EGCG, recoupling was independent of the mutation type when tested with uncoupled thin filaments containing TPM1 E54K, ACTC E99K, or TNNC1 G159D mutations as indicated in Table 1. It is interesting to note that when Ca^2+^ sensitivities of the phosphorylated and unphosphorylated troponin of re-coupled systems were measured, the shift in Ca^2+^-sensitivity was restored to a value close to the shift in Ca^2+^-sensitivity previously observed in PKA-phosphorylated native thin filaments. (see Figure 3 and Table S1). Following this preliminary screening, selected compounds were further investigated.

### There is no correlation between re-couplers and Hsp90 inhibitors

Although many of the compounds investigated were selected from a study on Hsp90 inhibitors, there does not seem to be any correlation between their efficacy as re-couplers and Hsp90 inhibition. Thus, for the EGCG analogs tested (**1–28**) there were four compounds that were both Hsp90 inhibitors and re-couplers, four that were re-couplers only, four that were Hsp90 inhibitors only and seven that were inactive in both assays (Table 1).

### The Ca^2+^-desensitizer and re-coupling activities may be separated

Although the lead compound, EGCG, was both a desensitizer and a re-coupler, we found that most of the compounds we studied did not have both properties. Some were desensitizers alone but the majority were re-couplers that did not influence myofilament Ca^2+^-sensitivity in WT muscle. Thus, the two activities of EGCG are not intrinsically linked.

**Figure 4:**
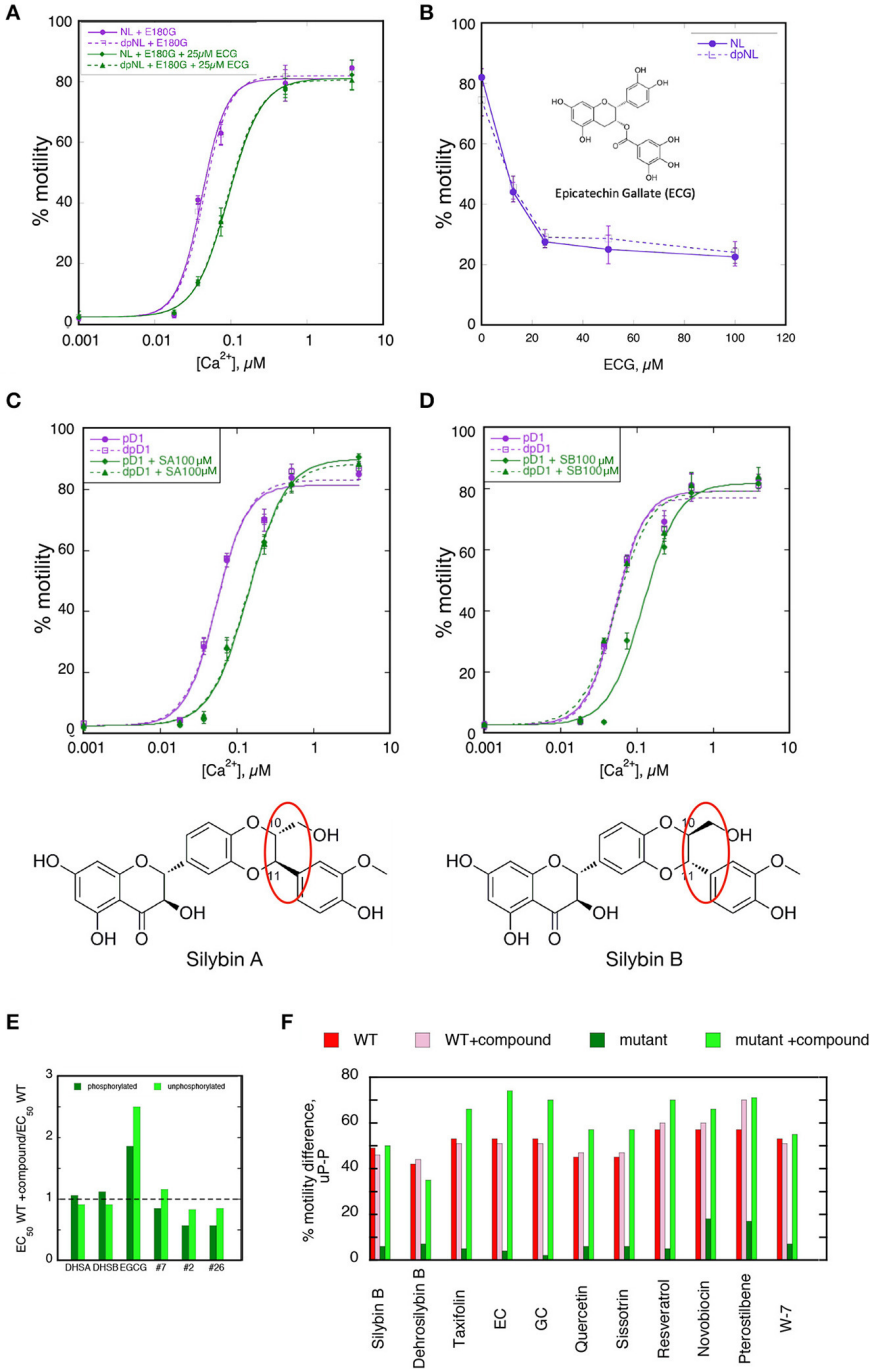
Separation of desensitization and recoupling activities **(A,B)** Epicatechin gallate (ECG) is a desensitizer but not a recoupler. **(A)** Ca^2+^-activation curves for phosphorylated and unphosphorylated thin filaments containing E180G tropomyosin in the presence and absence of 25 μM ECG. Solid lines, phosphorylated; dotted lines, unphosphorylated. Purple lines and points, E180G thin filaments; green lines and points, E180G thin filaments + ECG. **(B)** Dose-response curve for ECG activity measured at a constant Ca^2+^ concentration of 0.037. **(C,D)** Silybin A is a pure desensitizer whilst Silybin B is a pure recoupler Ca^2+^ activation curves are shown for phosphorylated and unphosphorylated thin filaments containing TnC G159D (DCM mutation) that are uncoupled. **(C)** In presence and absence of silybin A, **(D)** in presence and absence of Silybin B. Solid lines, phosphorylated; dotted lines, unphosphorylated. Purple lines and points, E180G thin filaments; green lines and points, E180G thin filaments + Silybin A or B. Structures of Silybin A and B are shown below the plots. **(E,F)** Compounds that recouple but are not Ca^2+^-desensitizers. **(E)** The effect of compounds on Ca^2+^-sensitivity of wild-type thin filaments. The ratio of EC_50_ in the presence and absence of tested compounds is plotted for the phosphorylated (dark green) and unphosphorylated (light green) states. Only EGCG is a significant desensitizer. DHSA/B, Dehydrosilybin A/B. **(F)** The effect of 100 μM drug treatment on the difference in fraction motile between phosphorylated and unphosphorylated states at 0.037μM Ca^2+^ in the phosphorylated and unphosphorylated states (see Figures 2C,D). Red bars, WT troponin; pink bars, WT troponin + compound. Dark green bars, E180G thin filaments, light green bars, E180G thin filaments + compound. Wild-type difference is unaffected by compound (not a desensitizer), whilst the value in E180G thin filaments is low indicating uncoupling, but restored by the compound indicating re-coupling.

Epicatechin gallate (ECG) is an example of a compound that is a pure desensitizer as illustrated in Figures 4A,B. When added to thin filaments that contain the uncoupling mutation E180G in tropomyosin, ECG equally reduces the Ca^2+^-sensitivity of both phosphorylated and unphosphorylated thin filaments so the system remains uncoupled. Silybin A also acts as a pure desensitizer (Figure 4C, Figure S2B).

Many of the compounds investigated were pure re-couplers. This is best demonstrated by their inability to influence the Ca^2+^-sensitivity of native thin filaments in either the phosphorylated or unphosphorylated state, in contrast to EGCG (Figure 4E). In mutant-uncoupled thin filaments, the pure recoupling ability is shown by the decrease in Ca^2+^-sensitivity of phosphorylated but not the unphosphorylated mutant thin filament in the presence of the compound as shown for Silybin B in Figure 4D, Quercetin in Figure 2A and Dehydrosilybin B in the Figure S3. In the rapid single Ca^2+^ concentration assay a large difference between % motility in the phosphorylated and unphosphorylated states is found in fully coupled systems. In wild-type thin filaments the difference is unaffected by the compounds indicating that the compound does not change wild-type Ca^2+^-sensitivity, whilst the value in E180G thin filaments is low, indicating uncoupling, but is restored by the compounds, indicating re-coupling.

Figure 4F illustrates this behavior for 10 compounds. We did not identify any compound apart from EGCG that was both a desensitizer and a re-coupler and 15 out of 40 compounds had no desensitizing or recoupling effects.

### Minimal effective structure

**Figure 5:**
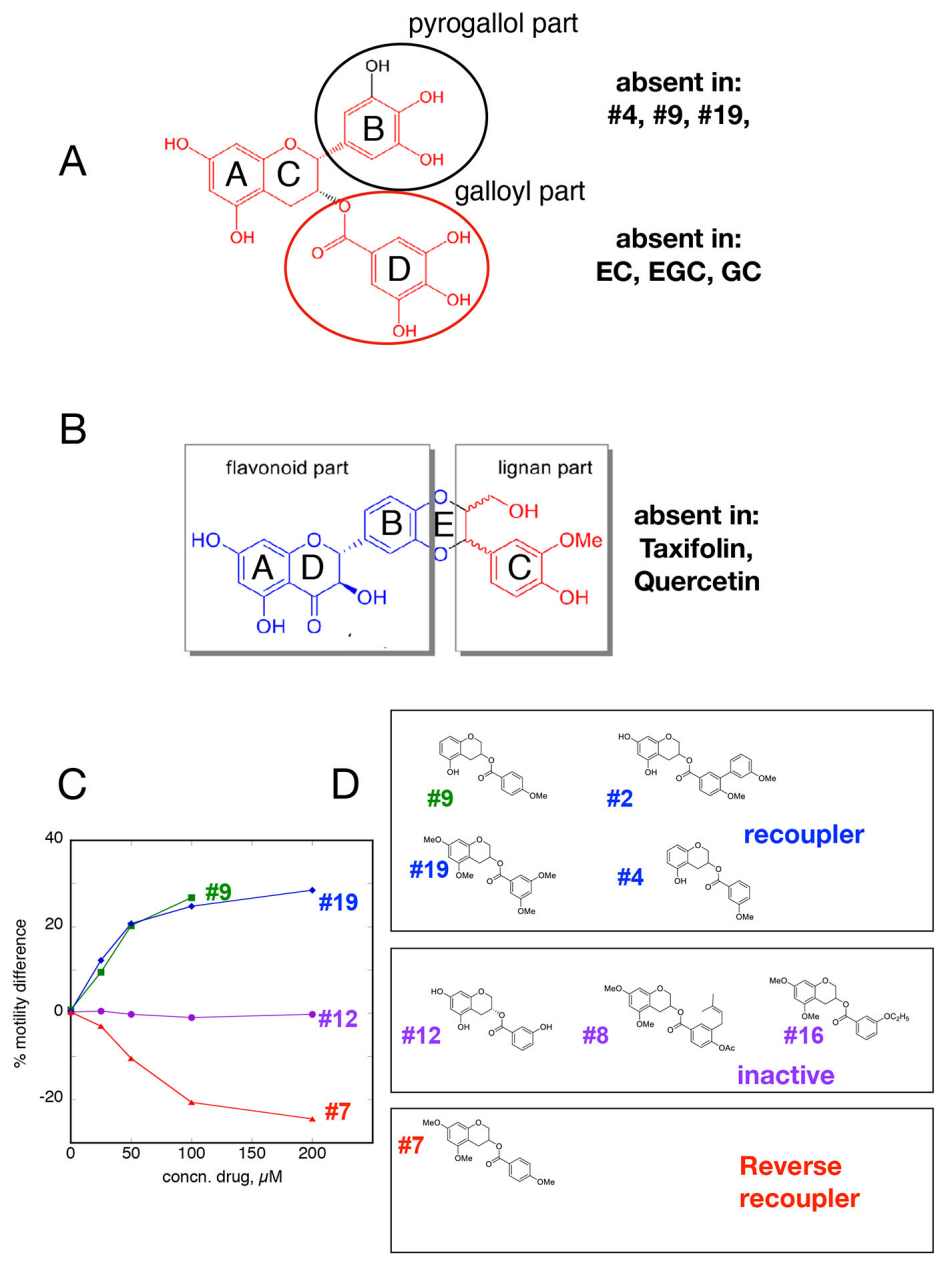
Structure-function relationships **(A,B)** *Minimal structures that re-couple*. **(C,D)** Small changes in A and D ring of EGCG determine recoupling activity **(C)**, Dose-response curves for re-couplers #9 and #19, inactive compound #12 and reverse re-coupler #7. **(D)** Structures of related re-coupling, inactive, and reverse recoupling compounds.

Since both EGCG and Silybin are quite large molecules, we investigated whether re-coupling could be achieved with smaller molecules. We found that removal of the pyrogallol ester moiety or the galloyl moiety, but not both, had no effect on re-coupling activity. Similarly, in Silybin it was possible to remove the lignan part without altering the compound's activity; thus, a substituted chromanone or chromane ring plus a phenolic ring seem to be sufficient for re-coupling (Figures 5A,B). We also observed that simpler structures such as the two-ring molecules, e.g. Resveratrol, Pterostilbene, compound #**2** (Figure 5), and even the single ring calmodulin antagonist W-7 were effective re-couplers (Figure 2, Table 1). This suggests that it is not the chromane/chromanone ring system that is essential but rather the substitutions that define the charge structure in ring A.

### Small differences change function

It is remarkable that the re-coupling function can be lost with small changes in key parts of the structure. For instance the change of an OH to H in the D ring of EGCG is sufficient to abolish recoupling whilst preserving desensitization (ECG, Figure 4A). The stereoisomers of Silybin and Dehydrosilybin, which have four or two chiral centers, respectively, have quite different activities. The B isoforms are good re-couplers contrary to the A isoforms (Figures 2, 4D, Figures S2B, S3).

A group of compounds with substitutions containing the core rings A, C, and D of EGCG shift function from good recoupling to no effect and even to reverse re-coupling. The structural differences are small, for instance compound **19** (re-coupler) and compound **7** that has a reverse effect differ only by the number and positions of *O*-methyl groups in the D ring whilst other substitutions in the D ring (compounds **8** and **16**) render the compound inactive. In addition, a number of substitutions in the A ring are compatible with recoupling when combined with an appropriate substitution in the D ring (compounds **9, 19**, and **2**) (Figures 5C,D). Methylation can alter properties of compounds since the phenolic OH is a weak acid and it is thus available as H-bond acceptor; OMe is also an H-bond acceptor but rather weaker and the acidity is lost. The position of free OH on the phenolic ring is likely to be important; for instance there is a huge difference in the reactivity of OHs in the *ortho(para*) and *meta* positions.

## Discussion

Mutations in contractile proteins can cause familial HCM or familial DCM. HCM has been linked to a higher myofilament Ca^2+^-sensitivity. In addition, we have identified a molecular level dysfunction common to both HCM and DCM-causing mutations (Papadaki et al., 2015; Messer et al., 2016). This is an uncoupling of the relationship between TnI phosphorylation and modulation of myofilament Ca^2+^-sensitivity, essential for normal responses to adrenaline. As a consequence, adrenergic response is blunted *in vivo*, which predisposes to heart failure under stress (Messer and Marston, 2014; Wilkinson et al., 2015). Thus uncoupling at the contractile protein level contributes to the symptoms of HCM and DCM and restoration of coupling is a significant target for therapy (Tardiff et al., 2015). We have identified compounds that can specifically reverse these abnormalities *in vitro* and therefore have potential for treatment that we call “re-couplers.” The first compound studied was Epigallocatechin-3-gallate (EGCG) (Tadano et al., 2010). EGCG is capable of both Ca^2+^ desensitization and re-coupling in mutant thin filaments (Papadaki et al., 2015). We have now identified a further 23 structural analogs that are capable of restoring coupling *in vitro*; many of these can re-couple independent of Ca^2+^-desensitization.

### Mechanism of coupling, uncoupling, and re-coupling

The normal change in myofilament Ca^2+^-sensitivity due to TnI phosphorylation by PKA is reported to be 2.00 fold ± 0.24 (EC_50_ P/unP, see Marston, 2016). It is remarkable that our current measurements show the same Ca^2+^ sensitivity shift in phosphorylated and unphosphorylated mutant-containing thin filaments when re-coupled (mean EC_50_ P/unP 2.2 ± 0.1 (*n* = 19) (Figure 3). This indicates a complete restoration of Ca^2+^ sensitivity modulation by TnI phosphorylation that is independent of the nature of the re-coupling compound and independent of the cardiomyopathy-related mutation (Figure 1). Therefore, it is reasonable to propose that these perturbations act by changing the equilibrium between a small number of pre-existing states.

**Figure 6:**
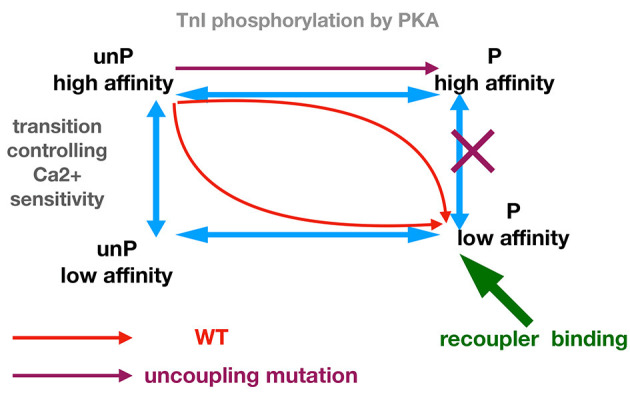
Allosteric model for modulation of Ca^2+^ -sensitivity by phosphorylation, mutations, and small molecule recouplers.

We propose that troponin exists in four functional states: phosphorylated form with high and low Ca^2+^-sensitivity and unphosphorylated form with high and low Ca^2+^ sensitivity (Figure 6). This model can account for the effects of TnI phosphorylation in wild-type and mutant (uncoupled) thin filaments and also the effects of re-coupling compounds. In wild-type muscle, phosphorylation results in a transition from the unphosphorylated, high Ca^2+^ sensitivity state to the phosphorylated low Ca^2+^ sensitivity state (red arrows). Mutations that produce uncoupling can only undergo transition from the unphosphorylated high Ca^2+^ sensitivity to phosphorylated high Ca^2+^ sensitivity state and the further transition to low Ca^2+^ sensitivity is blocked. Dvornikov et al. have proposed structural evidence that a normal phosphorylation-dependent structural transition is blocked in the TnI R145W HCM mutant troponin (Dvornikov et al., 2016). It is remarkable that the uncoupling phenomena can be induced by a wide range of mutations and other perturbations (Figure 1) and this suggests that the phosphorylated low affinity state is rather unstable and the high affinity state may be a default state.

The functions of small molecules can be accounted for by their preferential binding to, and hence stabilizing of, one or more troponin states. Thus, a small molecule could re-couple by preferentially binding to the phosphorylated low affinity state, which would overcome the blocked transition from phosphorylated high affinity state to low affinity phosphorylated state. In such a mechanism the small molecule would have no effect upon the transitions of wild-type troponin, as observed. Molecules that desensitize mutant thin filaments without re-coupling (ECG, Silybin A) could be accounted for if they bound equally to the phosphorylated and unphosphorylated low affinity states. The reverse re-coupler, compound #**7**, could be incorporated into this scheme if it is preferentially bound to the unphosphorylated low Ca^2+^ affinity state. Such predictions could be tested if ligand binding to wild-type and mutant troponin in the different states could be measured; in the absence of such techniques, molecular dynamics simulations of the ligands bound in phosphorylated and unphosphorylated states would be a suitable approach (Zamora et al., 2017).

### Troponin structural correlations

Although structural determinations of ligand binding (EGCG, W-7) to troponin subunits have been made, most studies have involved binary complexes (TnI and TnC) and partial sequences (Robertson et al., 2010; Botten et al., 2013; Hwang and Sykes, 2015). Recent molecular dynamics simulations have emphasized the importance of studying the intact troponin core with TnI, TnC, and TnT present (Papadaki and Marston, 2016; Zamora et al., 2016). Moreover, studies on the docking of EGCG, Silybin A and Silybin B by molecular dynamics simulations suggest that these compounds bind and crosslink the N-terminal peptide of TnI in the vicinity of the phosphorylatable serines22 and−23 to the N terminal domain of TnC and that the three compounds have different dynamics. In this position the re-coupling compound could influence the phosphorylation-dependent modulation of Ca^2+^-sensitivity of TnC compatible with our four state model (Zamora et al., 2017).

It is notable that the phosphorylatable N-terminus and the “switch peptide” of TnI, critical for Ca^2+^-regulation, are intrinsically disordered peptides (Hwang et al., 2014; Papadaki and Marston, 2016). By analogy with similar disordered peptides containing regulatory phosphorylation sites it is suggested that there may be negligible differences in time-averaged structures between phosphorylated and unphosphorylated states, but rather phosphorylation induces a change in protein dynamics from an ordered to a less ordered state (Colson et al., 2012).

More precise structure-function analysis requires further extensive docking modeling, however, it appears that the substituted chromane (in EGCG) or chromanone (in Silybin) are fundamental. The way in which the substitutions define the structure, charge distribution, hydrogen bonding potential or acidity of the A ring, apparently involved in its intercalation between TnI and TnC, is likely to be critical for the compound effect. In the EGCG analogs it seems that either the B ring (pyrogallol) or the D ring (galloyl) are sufficient to confer re-coupling activity, so one ring may be able to assume the configuration of the other when one of them is absent. In Silybin and Dehydrosilybin, the lignan part is not fully needed for function (taxifolin, quercetin) and only the B isomers are recouplers, whilst the A stereoisomers of Silybin and Dehydrosilybin are inactive as recouplers. In this case it seems likely that the A isomers have an unfavorable conformation that prevents the A and D rings binding in the same way as the B isoform. It is noteworthy that the B isomer is more active than the A isomer is several different bio-assays (Kren and Walterová, 2005; Biedermann et al., 2014).

### Physiological correlates

The main effect of uncoupling of the modulation of Ca^2+^ sensitivity by TnI phosphorylation is the loss of the lusitropic response to beta adrenergic stimulation, which is usually accompanied by blunting of the whole physiological adrenergic response. This is demonstrated most directly by a lack of response to dobutamine stimulation observed at the whole animal, papillary muscle and single cardiomyocyte levels (Song et al., 2010, 2011; Wilkinson et al., 2015). Tadano showed that EGCG restored the impaired pump function due to diastolic dysfunction in a transgenic HCM mouse model (Tadano et al., 2010). Our preliminary experiments in isolated cardiomyocytes from ACTC E99K HCM transgenic and non-transgenic mice (Marston et al., 2018) show that the lusitropic effect of dobutamine is blunted in ACTC E99K mouse but could be restored by Resveratrol and Silybin B, whilst the effect of EGCG is anomalous, reflecting the known off target effects, especially positive inotropy (Feng et al., 2012). Further structural and functional investigations of these compounds could produce a new lead compound to direct our search for an effective and specific re-coupler with a practical potential in the treatment of inherited cardiomyopathies at the fundamental level.

## Author contributions

AS, AM, MP, AC performed the experiments reported here; BB, AK, VK, DB prepared and supplied EGCG and silybin analogs; SM obtained funding, directed research and wrote the paper. All the authors contributed to editing and completing the paper.

### Conflict of interest statement

The authors declare that the research was conducted in the absence of any commercial or financial relationships that could be construed as a potential conflict of interest.
