# Supplementary material for: Molecular Defects in Cardiac Myofilament Ca2+-Regulation Due to Cardiomyopathy-Linked Mutations Can Be Reversed by Small Molecules Binding to Troponin
Source: Front Physiol. 2018 Mar 27;9:243. doi: 10.3389/fphys.2018.00243 (PMC5881522; doi:10.3389/fphys.2018.00243)
Supplement: Supplementary file 1 [file DataSheet1.PDF]

Supplementary Material

Molecular defects in cardiac myofilament Ca^{2+}-regulation leading to hypertrophic cardiomyopathy can be reversed by small molecules binding to troponin

Alice Sheehan[1], Andrew Messer[1], Maria Papadaki[1], Afnan Choudhry[1], Vladimír Křen[2], David Biedermann[2], Brian Blagg[3], Anuj Kandelwahl[3], Steven Marston[1]

[1] NHLI, Imperial College London, Du Cane Road, London W12 0NN, UK

[2] Institute of Microbiology of the Czech Academy of Sciences, Laboratory of Biotransformation, Vídeňská 1083, 14220 Prague, Czech Republic

[3] The University of Kansas, 1251 Wescoe Hall Drive, Malott 4070, Lawrence, KS 66045-7562, USA

Figure S1 Structures of the compounds used in this study

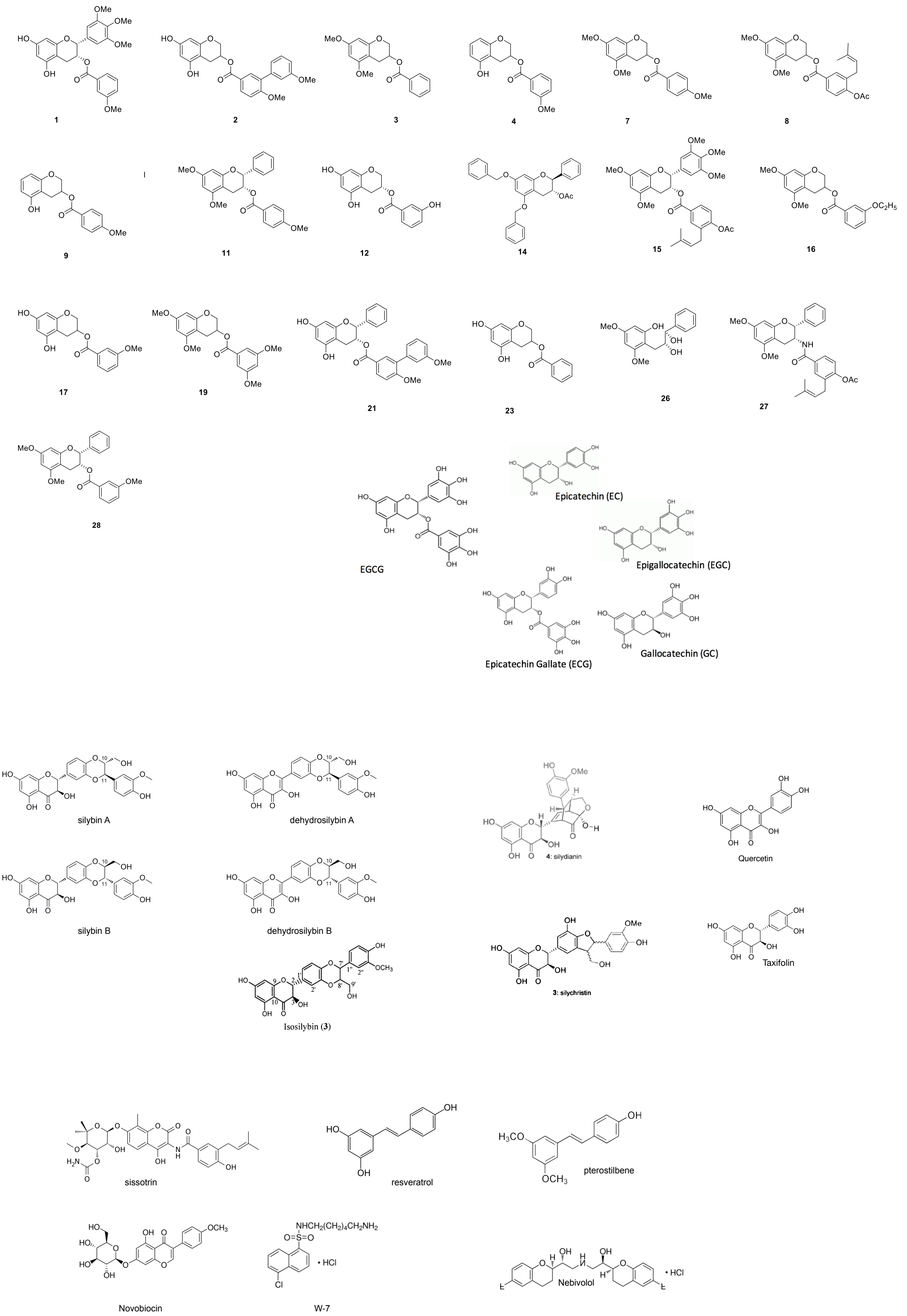

Table S1

EC_{50} for Ca^{2+} activation of phosphorylated and unphosphorylated thin filaments incorporating troponin from HCM and DCM tissue samples that are uncoupled was measured by *in vitro* motility assay in the presence and absence of 100 µM EGCG. Mutations have been identified in cardiac troponin C (TNNC1 gene), troponin I (TNNI3), tropomyosin 1.1 [1] (TPM1), actin (ACTC) and myosin binding protein-C (MYBPC3). Tissue samples came from human, mouse and cat hearts. Also included are two samples from cats with HCM and three human samples obtained by septal myectomy with HCM diagnosis but no identified mutation found after genotyping. EGCG restored coupling to all the uncoupled systems. Data from [2-4]

Table S1

phenotype	mutation	EC_{50} phosphorylated	EC_{50} unphoshorylated	EC_{50} phosphorylated + EGCG	EC_{50} unphosphorylated + EGCG	**EC_{50} P/unP**	**EC_{50} P/unP +EGCG**
Wild type		0.14±0.03 (6)	0.059±0.011 (6)	0.25±0.03 (6)	0.15±0.02 (6)	2.4	1.7
DCM							
	TNNC1 G159D	0.092±0.004 (5)	0.095±0.0005 (5)	0.19±0.03 (5)	0.088±0.005 (5)	0.97	2.2
	TPM1 E40K	0.17±0,05 (3)	0.16±0.05 (3)	0.26±0.04 (3)	0.058±0.02 (3)	1.02	4.0
	TPM1 E54K	0.11±0.013 (5)	0.11±0.02 (5)	0.23±0.03 (5)	0.071±0.005 (5)	0.95	3.1
	ACTC E361G	0.087±0.002 (5)	0.080±0.002 (5)	0.20±0.02 (5)	0.081±0.002 (5)	1.08	2.3
	TNNI3 K36Q	0.077±0.006 (3)	0.07±0.011 (3)	0.18±0.03 (3)	0.088±0.01 (3)	1.02	1.9
HCM							
	TPM1 E180G	0.086±0.002 (5)	0.087±0.11 (3)	0.12±0.02 (3)	0.043±0.001 (3)	0.98	2.3
	MBPC3 R820W	0.047±0.002 (4)	0.049±0.002 (4)	0.097±0.007 (4)	0.044±0.001 (4)	0.97	2.0
	ACTC E99K	0.074±0.005 (5)	0.074±0.005 (5)	0.21±0.03 (5)	0.10±0.007 (5)	0.99	2.0
	TNNT2 R92Q	0.03±0.004	0.003±0.004	0.137±0.044	0.048±0.006	1.1	2.8
	TNNT2 K280N	0.11±0.012(3)	0.097±0.004 (3)	0.23±0.03 (3)	0.11±0.005 (3)	1.08	2.3
HCM mutation unknown							
	cat H13	0.030	0.029	0.068	0.035	1.03	1.9
	cat H14	0.039	0.046	0.048	0.037	0.85	1.3
	myectomy MR	0.041 (2)	0.041 (2)	0.073 (2)	0.043 (2)	0.98	1.7
	myectomy MV	0.053±0.004 (3)	0.054±0.003 (3)	0.111±0.008 (3)	0.054±0.004 (3)	1.00	2.1
	myectomy MD	0.054±0.001 (3)	0.053±0.001 (3)	0.111±0.002 (3)	0.041±0.001 (3)	1.03	2.7

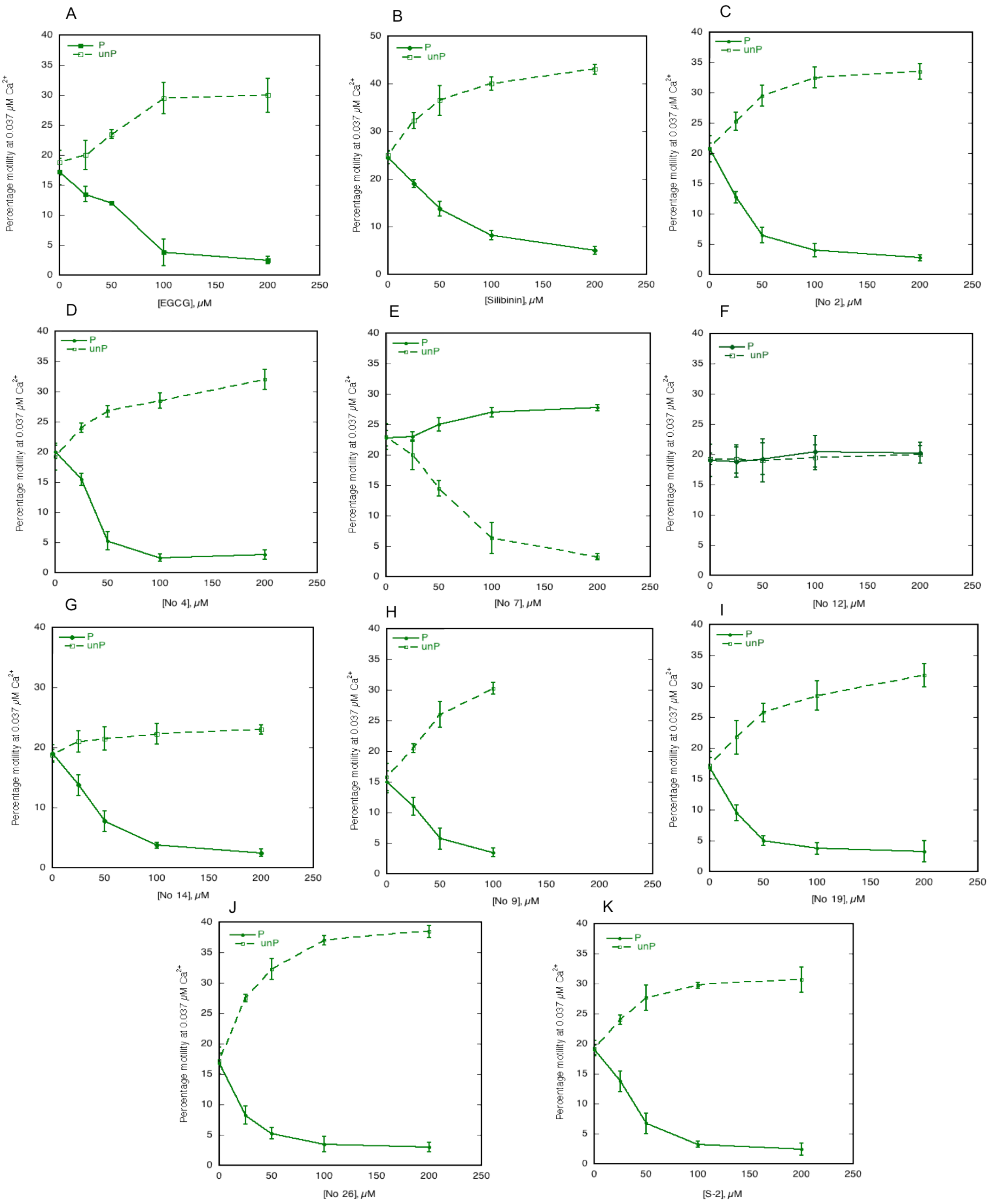

Figure S2A. Dose response curves for EGCG structural analogues

Percentage motility of phosphorylated or dephosphorylated E180G thin filaments was measured at a constant Ca^{2+} concentration of 0.037 μM with increasing drug doses. A) EGCG B) Silybin C) No 2 D) No 4 E) No 7 F) No 12 G) No 14 H) No 9 I) No 19 J) No 26 K) S-2. The drug compounds are in order of the screening experiments performed.

: Dose response data summary for percentage motility difference between phosphorylated and dephosphorylated *TPM1* E180G mutant thin filaments treated with different compounds

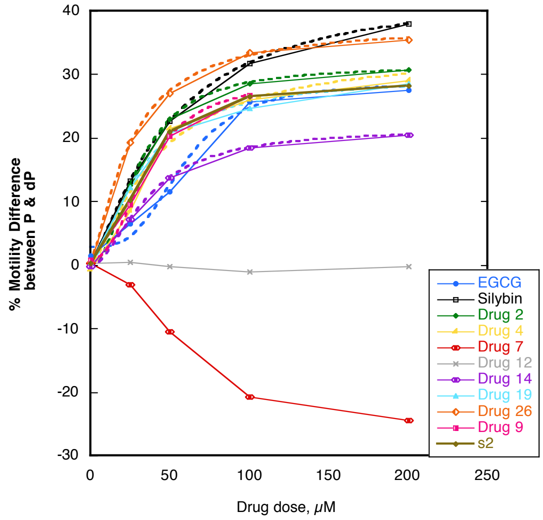

Percentage motility difference of phosphorylated and dephosphorylated thin filaments treated with different compounds. Percentage motility was measured at 0.037 μM Ca^{2+} and the Hill equation was fitted to the data to create the curves (dotted lines). Different colored lines represent different compounds.

Data summary for the difference between phosphorylated and dephosphorylated *TPM1* E180G HCM mutant thin filaments treated with different compounds

E180G + Drug	EC_{50}, μM	n_H	Magnitute (% difference)
**EGCG**	58.83 ± 13.31	2.15 ± 0.89	30.23 ± 4.81
**Silibinin**	47.21 ± 2.66	1.3 ± 0.078	43.74 ± 1.29
**2**	30.5 ± 0.41	2.02 ± 0.067	31.31 ± 2.24
**4**	33.98 ± 2.13	2.69 ± 0.43	28.52 ± 1.05
**7**	67.05 ± 20.31	1.71 ± 0.57	28.16 ± 5.3
**9**	34.83 ± 2.91	2.22 ± 0.37	29.32 ± 1.9
**12**	No Effect	No Effect	No Effect
**14**	36.23 ± 0.82	1.81 ± 0.08	21.44 ± 0.29
**19**	30.79 ± 2.9	1.54 ± 0.3	29.68 ± 1.63
**26**	24.59 ± 1.49	1.37 ± 0.2	37.8 ± 1.4
**S-2**	32.24 ± 0.4	2.28 ± 0.071	28.57 ± 0.21

Figure S2B. Dose response curves for silybin stereomers and derivatives

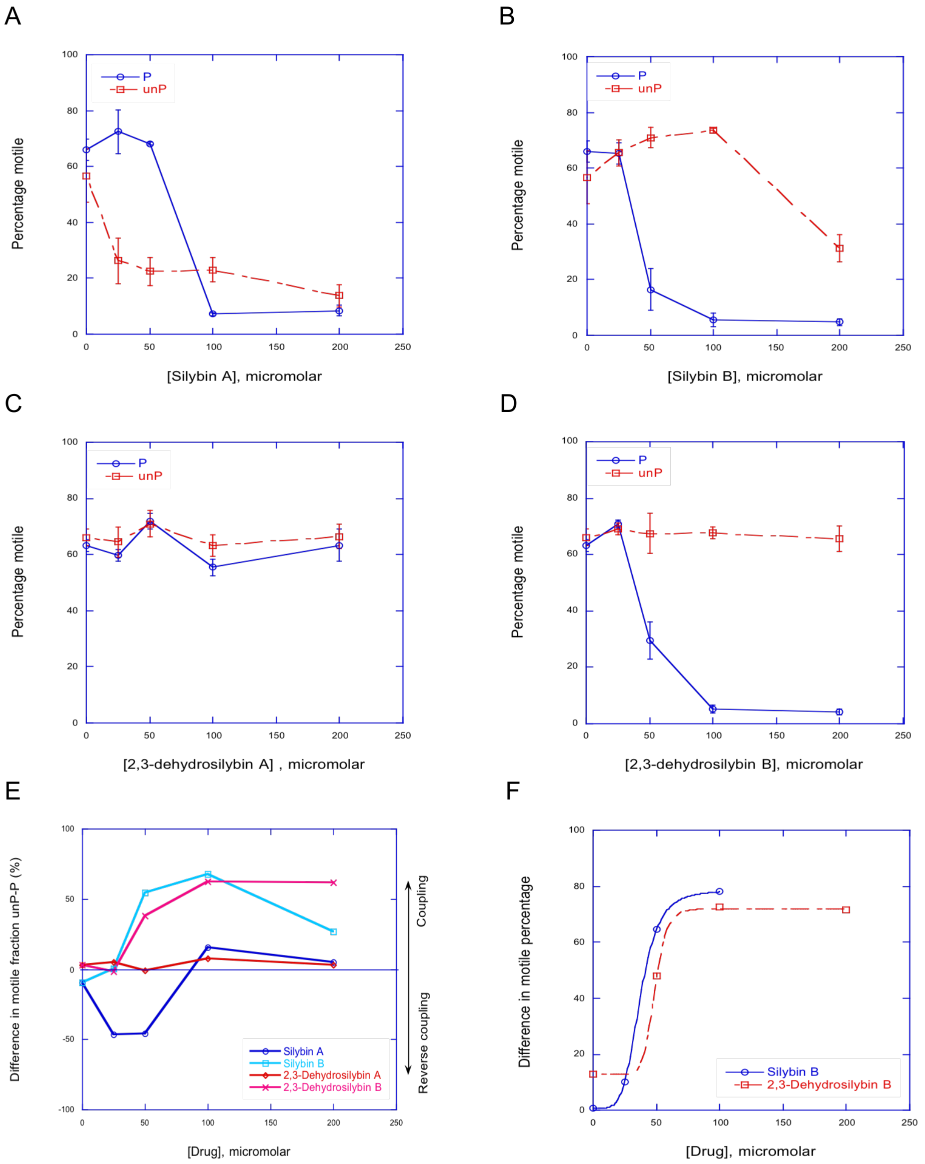

Each graph illustrates a single experiment. Error bars are derived from the standard error of the mean from the four recordings of each flow chamber. (A) Silybin A has a complex dose sensitive effect. Lower doses (25 and 50μM) reduce the unphosphorylated (unP) motile percentage while the phosphorylated (P) motile percentage remains unaltered. This apparent paradox where the phosphorylated state has a higher Ca^{2+}-sensitivity is termed reverse coupling. At higher doses the P motile percentage is reduced beyond unP, thus restoring normal coupling but the stunting of motility diminishes the magnitude of the effect. (B) Silybin B begins to recouple at doses of 50μM, peaking at 100μM, but reducing motility at higher doses. Recoupling is accomplished by reducing the Ca^{2+}-sensitivity of P thin filaments. (C) 2,3-dehydrosilybin A is a poor recoupling molecule. (D) 2,3-dehydrosilybin B restores coupling at similar doses to Silybin B, but motility is not reduced at higher doses. (E) The difference in motile percentage made by the phosphorylation state is plotted for all four molecules to compare the magnitude of their coupling effect at each dose. Positive values reflect normal coupling and negative values reflect reverse coupling. (F) The Hill equation is applied to the data for Silybin B and 2,3-dehydrosilybin B to yield potency (EC_{50})

Figure S3. Ca^{2+} activation curves in presence of pure dehydrosilybin isomers.

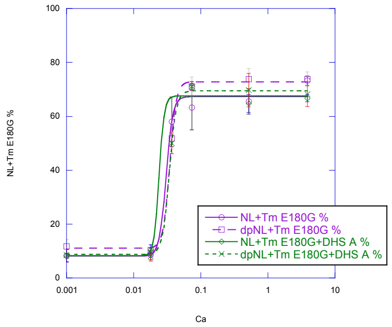

y = m1+(m2*m0^m3) / (m4^m3+m...	Value	Error
m1	8.0754	5.2352
m2	59.275	6.1141
m3	8.6722	17.077
m4	0.030513	0.011922
Chisq	54.883	NA
R	0.99374	NA

y = m1+(m2*m0^m3) / (m4^m3+m...	Value	Error
m1	11.108	1.7411
m2	61.713	2.2037
m3	8.9796	18.506
m4	0.034523	0.0050163
Chisq	6.5274	NA
R	0.99929	NA

y = m1+(m2*m0^m3) / (m4^m3+m...	Value	Error
m1	8.2504	4.7082
m2	59.333	5.7664
m3	11.596	31635
m4	0.024041	19.02
Chisq	22.168	NA
R	0.9973	NA

y = m1+(m2*m0^m3) / (m4^m3+m...	Value	Error
m1	8.8114	1.6935
m2	60.666	2.118
m3	8.4982	12.251
m4	0.034121	0.0040838
Chisq	6.0701	NA
R	0.99933	NA

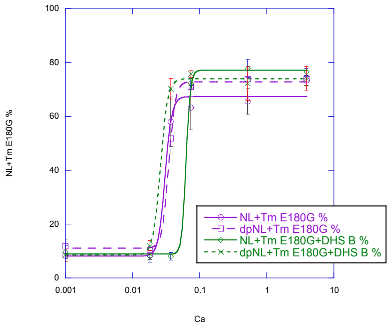

y = m1+(m2*m0^m3) / (m4^m3+m...	Value	Error
m1	8.0754	5.2352
m2	59.275	6.1141
m3	8.6722	17.077
m4	0.030513	0.011922
Chisq	54.883	NA
R	0.99374	NA

y = m1+(m2*m0^m3) / (m4^m3+m...	Value	Error
m1	11.108	1.7411
m2	61.713	2.2037
m3	8.9796	18.506
m4	0.034523	0.0050163
Chisq	6.5274	NA
R	0.99929	NA

y = m1+(m2*m0^m3) / (m4^m3+m...	Value	Error
m1	8.8762	1.0797
m2	68.249	1.5269
m3	14.06	66.576
m4	0.062741	0.048937
Chisq	4.6629	NA
R	0.99965	NA

y = m1+(m2*m0^m3) / (m4^m3+m...	Value	Error
m1	8.5077	1.7584
m2	65.493	2.0341
m3	7.8897	1.2648
m4	0.025927	0.0015557
Chisq	6.1841	NA
R	0.99941	NA

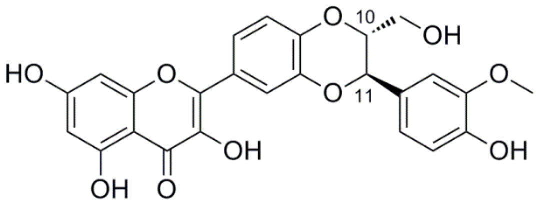

dehydrosilybin A

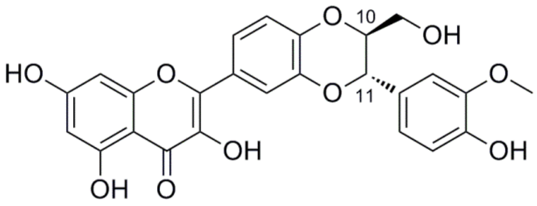

dehydrosilybin B

REFERENCES

1. Geeves, M.A., Hitchcock-DeGregori, S.E. & Gunning, P.W. A systematic nomenclature for mammalian tropomyosin isoforms. *J Muscle Res Cell Motil* **36**, 147-153 (2015).
2. Messer, A.*, et al.* Mutations in troponin T associated with Hypertrophic Cardiomyopathy increase Ca^{2+}-sensitivity and suppress the modulation of Ca2+-sensitivity by troponin I phosphorylation. *Arch Biochem Biophys* **601**, 113-120 (2016).
3. Papadaki, M., Vikhorev, P.G., Marston, S.B. & Messer, A.E. Uncoupling of myofilament Ca^{2+} sensitivity from troponin I phosphorylation by mutations can be reversed by epigallocatechin-3-gallate. *Cardiovasc Res* **108**, 99-110 (2015).
4. Messer, A.E.*, et al.* Investigations into the Sarcomeric Protein and Ca^{2+}-Regulation Abnormalities Underlying Hypertrophic Cardiomyopathy in Cats (*Felix catus*). *Front Physiol* **8**, 348 (2017).
